# Transition matrices model as a way to better understand and predict intra-hospital pathways of covid-19 patients

**DOI:** 10.1038/s41598-022-22227-8

**Published:** 2022-10-20

**Authors:** Arnaud Foucrier, Jules Perrio, Johann Grisel, Pascal Crépey, Etienne Gayat, Antoine Vieillard-Baron, Frédéric Batteux, Tobias Gauss, Pierre Squara, Seak-Hy Lo, Matthias Wargon, Romain Hellmann

**Affiliations:** 1 Ile-de-France Regional Health Agency, Paris, France; 2 Hôpital Beaujon, DMU PARABOL, University of Paris, APHP, Paris, France; 3 Data Science and Analytics Department, SESAN, Paris, France; 4 Ecole des Hautes Etudes en Santé Publique, REPERES (Recherche en Pharmaco-Epidémiologie et Recours Aux Soins), University of Rennes, EA 7449, Rennes, France; 5 Department of Anaesthesiology and Critical Care, Hôpital Lariboisière, DMU PARABOL, University of Paris, Paris, France; 6 Inserm UMR-S 942, Cardiovascular Markers in Stress Conditions (MASCOT), University of Paris, Paris, France; 7 University Hospital Ambroise Paré, APHP, Boulogne-Billancourt, and Université de Versailles Saint Quentin en Yvelines UMR 1018, Boulogne-Billancourt, France; 8 Institut Cochin, INSERM U1016, Université Paris Descartes, Sorbonne Paris Cité, Paris, France; 9 Grenoble Alpes Trauma Centre, Pôle Anesthésie-Réanimation, CHU de Grenoble, La Tronche, France; 10 Department of Intensive Care and Cardiology, Clinique Ambroise Paré, Neuilly-Sur-Seine, France; 11 Emergency Department, Hôpital Delafontaine, Saint-Denis, France; 12 Observatoire Regional Des Soins Non Programmés-Ile-de-France, Saint-Denis, France; 13 Emergency Department, Bichat Hospital, Assistance Publique-Hôpitaux de Paris, Paris, France

**Keywords:** Viral infection, Health services, Public health

## Abstract

Since January 2020, the SARS-CoV-2 pandemic has severely affected hospital systems worldwide. In Europe, the first 3 epidemic waves (periods) have been the most severe in terms of number of infected and hospitalized patients. There are several descriptions of the demographic and clinical profiles of patients with COVID-19, but few studies of their hospital pathways. We used transition matrices, constructed from Markov chains, to illustrate the transition probabilities between different hospital wards for 90,834 patients between March 2020 and July 2021 managed in Paris area. We identified 3 epidemic periods (waves) during which the number of hospitalized patients was significantly high. Between the 3 periods, the main differences observed were: direct admission to ICU, from 14 to 18%, mortality from ICU, from 28 to 24%, length of stay (alive patients), from 9 to 7 days from CH and from 18 to 10 days from ICU. The proportion of patients transferred from CH to ICU remained stable. Understanding hospital pathways of patients is crucial to better monitor and anticipate the impact of SARS-CoV-2 pandemic on health system.

## Introduction

The SARS-CoV-2 pandemic has affected the whole of Europe. In France, the impact has been different depending on the geographical area. To date, three periods have been particularly significant in terms of the influx of patients into the healthcare system: spring 2020, fall-winter 2020 and spring 2021, corresponding to three "epidemic waves". Scientific and medical progress made throughout 2020 and 2021 has led to changes in the management of these patients. Recently, a report analyzed 110,000 patients hospitalized in conventional hospitalization or critical care for COVID-19 reasons between March 1 and June 1, 2020, (first wave) and around 200,000 patients between September 1, 2020, and February 1, 2021, in the whole of France^1^. The results of this work show that some regions were more severely impacted than others. For example, the mortality rate observed in the Paris region was higher than in other areas.

Several cohort studies have identified risk factors for severe forms of COVID-19 and have described the profile of hospital stays related to this new disease. The cohort study conducted on more than 20,000 patients hospitalized in Great Britain during the first quarter of 2020 showed an overall mortality rate of around 25% and the need for intensive care in 17% of cases^2^. These data are consistent with the previous data, in particular those from the first descriptions in China^3^.

Although many demographic descriptions are now available, few data cover the modalities of hospitalization, particularly intra-hospital pathways. In addition, there is little data comparing the characteristics between the different epidemic waves observed in Europe and, more specifically, in France. In such pandemics, when healthcare systems are under extreme strain, any change in the management response has a strong impact on bed occupancy, caregiver mobilization and resource allocation, including the need for ephemeral intensive care units. Understanding and anticipating these changes may help quickly reallocate resources to standard care when virus circulation declines. The main objective of this study was to compare the hospital pathways during these three periods, with the hypothesis that the demographic characteristics of hospitalized patients, the intra-hospital pathways and the impact on the healthcare system were different during the three epidemic waves observed in the Ile-de-France (Paris area). This hypothesis was supported by the emergence of new variants whose contagion and virulence levels were believed to be different.

## Material and methods

To make the comparison between epidemic waves as homogeneous as possible, we selected the characteristics of the care pathway through all of the epidemic waves from March 1, 2020, to July 19, 2021, (described as periods).The ascending phase of each wave was defined as the period between the beginning of the admissions surge and the peak of hospital admissions. The starting point of each period corresponds to the lowest point of hospital admission between two periods. The peak point of each period corresponds to the highest hospital admission in the considered period. The end of a period (and the beginning of the next period) was defined by the lowest admissions value between two consecutive periods.

### Data

SIVIC (*Système d’information pour le suivi des victimes d’attentats et de situations sanitaires exceptionnelles*) is a French national database created to keep track of casualties from terrorist attacks. Since 2015, its scope has been extended to include the surveillance of exceptional sanitary situations. It was thus used from mid-March 2020 to register data relative to patients infected with COVID-19. Only Paris-area data were analyzed.

The portal is accessible to identified users from different entities: hospitals, ambulance service (Service d’Aide Médicale Urgente—SAMU), regional health agencies (Agence Régionale de Santé-ARS), etc. It is part of a broader crisis management information system covering the complete patient pathway allowing real-time follow-up of healthcare capacities.

SIVIC data used in our study includes anonymized patient information, current patient status (hospitalized, deceased or returned home), type of clinical care received (conventional hospitalization, intensive care, rehabilitation and recuperative care, or psychiatric hospitalization), date of admission into each of the mentioned services, age, gender, hospital name and department. We are thus able to infer patient transfers.

To focus on adults patients, we removed patients under 16 years old, patients whose status was updated several times a day; pathways solely comprised of either return home, rehabilitation and recuperative care, or death; pathways starting with either rehabilitation and recuperative care or psychiatric hospitalization; and emergency department (ED) visits.

### Ethical and legal considerations

The study was based on a secondary use of a database/pseudonymised data collected from health professionals.

The purpose of study is the epidemiological surveillance at the regional level since the article 11 of the Law n° 2020-546 of May 11, 2020. This article extending the state of sanitary emergency, allows the regional health authorities (Agence régionale de santé-ARS) to adapt the existing information systems to fight against the propagation of the epidemic of COVID-19 and for the duration strictly necessary to this objective or, at the latest, until July 31, 2022. Article 14 of Decree No. 2020-551 of May 12, 2020 on the information systems mentioned in Article 11 of Law No. 2020-546 of May 11, 2020 extending the state of health emergency already authorizes the ARS "to implement processing of personal data for the following purposes: […] 3° Epidemiological surveillance at the regional level."

In health crisis, SIVIC is a system that has not been submitted to the ethic review board because it does not have the ambition to be permanent. It is a victim management tool.

All methods were carried out in accordance with relevant guidelines and regulations.

### Analysis of transition dynamics

A Markov chain is a sequence of random variables that allows us to model the dynamic evolution of a random system. The fundamental property of Markov chains, known as the Markov property, is that their future evolution depends on the past only through their current state. Markov-based models (Markov chains and their derivatives, such as hidden Markov chains and semi-Markov chains) have been used in the literature to model clinical pathways for specific diseases like schizophrenia^4^ or chronic kidney disease^5^ with a focus on improving patient care, for cost–benefit analysis of different treatments, for example^6^. Markov chains have also been used to support medium- and short-term resource planning.^4^

This type of model thus allows us to model the intra-hospital patient pathway as a succession of states with a length of stay in each state and transition frequencies from one state to another.

We consider the states for COVID-19 patients as either presence in a particular type of ward or an end state such as death or recovery (discharge). Due to the granularity of the data describing patients, we have two types of ward: conventional hospitalization (CH) and intensive care unit (ICU). Whereas a formal Markov model would describe length of stay in one state following geometric distributions, we fit length of stay distributions empirically, hence working in a semi-Markovian framework.

For more details about the construction of the transition matrices, see Online Appendix S1.

We describe the data used and detail our semi-Markovian approach by explaining how we calculated the transition frequencies from one type of ward to another and how we were able to fit in-ward length-of-stay distributions (Online Appendix S1).

### Statistical analysis

We done the analysis of the evolution of the proportions presented in the transition matrices as follows. Assuming that the death of one patient is not linked to the death of another, which, given populations with numbers in the order of a thousand, the variables "the patient died" follow independent Bernoulli distributions of the same parameter (probability $p$). Thus, the sum of these variables follows a Binomial distribution of parameters (number of deceased patients $n$; probability $p$). In this case, the variance of the number of deaths is $np(1-p)$. Thus, under the given assumptions, the variable $F$ "frequency of occurrence of deaths" is such that $nF$ follows a Gaussian distribution of parameters ($np$; $\sqrt{np(1-p}$). The same reasoning applies to the CH, ICU and discharge cases as well as to the numbers of intraregional transfers, types of hospitalization, and outcomes, or for some opposite situations. For more details, see Online Appendix S1.

## Results

**Figure 1:**
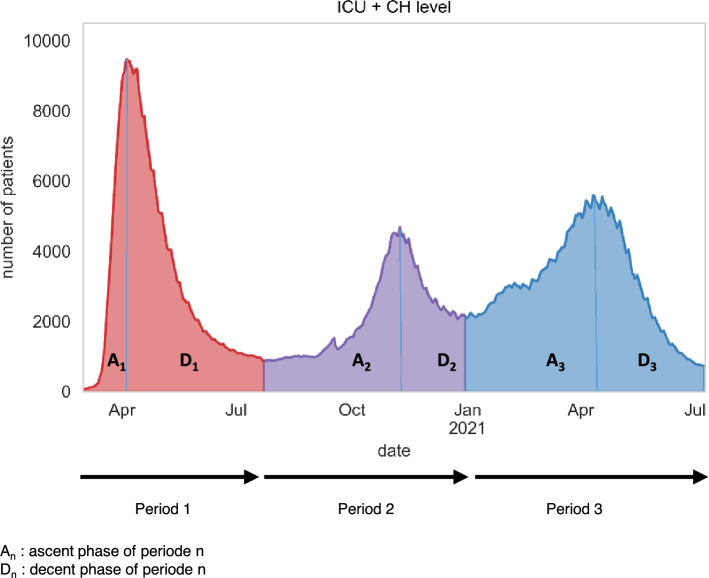
Evolution of the number of patients hospitalized in intensive care units (ICU) and conventional hospitalization (CH) sectors according to the three identified periods.

We identified three epidemic periods in the Paris area. The first period began on March 1, 2020, and ended on July 24, 2020, at which point the second period began, subsequently concluding on December 31. The third period began on December 31 and ran until July 19, 2021, where the analysis stops (see Fig. 1).

In total, we analyzed the care patterns of 90,834 patients: 27,727 in the first period; 23,683 in the second period; and 39,424 in the third period. The majority of patients were men, but this proportion decreased over time (from 58 to 54%). The median age was always above 65 years (68, 71, and 66 years, respectively). Around 80% of patients were managed in conventional hospitalization during the first period and 11.6% of patients had mixed management including conventional hospitalization and intensive care unit management. These proportions changed over time to 74.9% and 16.4% respectively.

The median length of stay (LOS) remained relatively stable in the conventional hospitalization sectors, at around 8 days, whereas the median length of stay decreased in the critical care sectors between the first and second periods (from 12 to 8 days) and remains stable between second and third period (8 and 9 days). The mortality rate statistically decreased regularly from 17.7 to 15.7% (Table 1).

**Table 1:** Typology of patients and main characteristics of care pathway and outcomes for each period.

	Period 1 (n = 27,727)	Period 2 (n = 23,683)	Period 3 (n = 39,424)
**Patients**			
Age, years	66.9 ± 17.5 68 (55–81)	68.1 ± 17.9* 71 (57–82)	64.8 ± 18.1^#‡^ 66 (53–79)
Men	15,930 (57.5%)	13,160 (55.6%)	21,262 (53.9%)
**Type of hospitalization**			
CH only	22,347 (80.6%)	18,737 (79.1%)*	29,513 (74.9%)^#‡^
ICU only	2164 (7.8%)	1805 (7.6%)	3445 (8.7%)
Both CH and ICU	3216 (11.6%)	3141 (13.3%)*	6466 (16.4%)^#‡^
**Length of stay**			
CH (days)	14.2 ± 23.9 8 (4–14)	10.7 ± 9.8* 8 (5–13)	9.5 ± 8.8^#‡^ 7 (4–12)
ICU (days)	20.0 ± 24.0 12 (6–25)	15.7 ± 18.4* 8 (5–19)	14.3 ± 15.8^#‡^ 9 (5–17)
**Number of intraregional transfers**			
	2907 (10.5%)	1411 (6.0%)*	3046 (7.7%)^#‡^
**Outcomes**			
Discharge	22,807 (82.3%)	19,861 (83.9%)*	33,248 (84.3%)^**‡**^
Death	4920 (17.7%)	3822 (16.1%)*	6176 (15.7%)^**‡**^

Data are expressed in mean ± sd and P50 (P25-P75).

*CH* conventional hospitalization, *ICU* intensive care unit, *LOS* length of stay.

*Significant difference between period 1 and period 2.

^#^Significant difference between period 2 and period 3.

^‡^Significant difference between period 1 and period 3.

### Transition matrices

**Figure 2:**
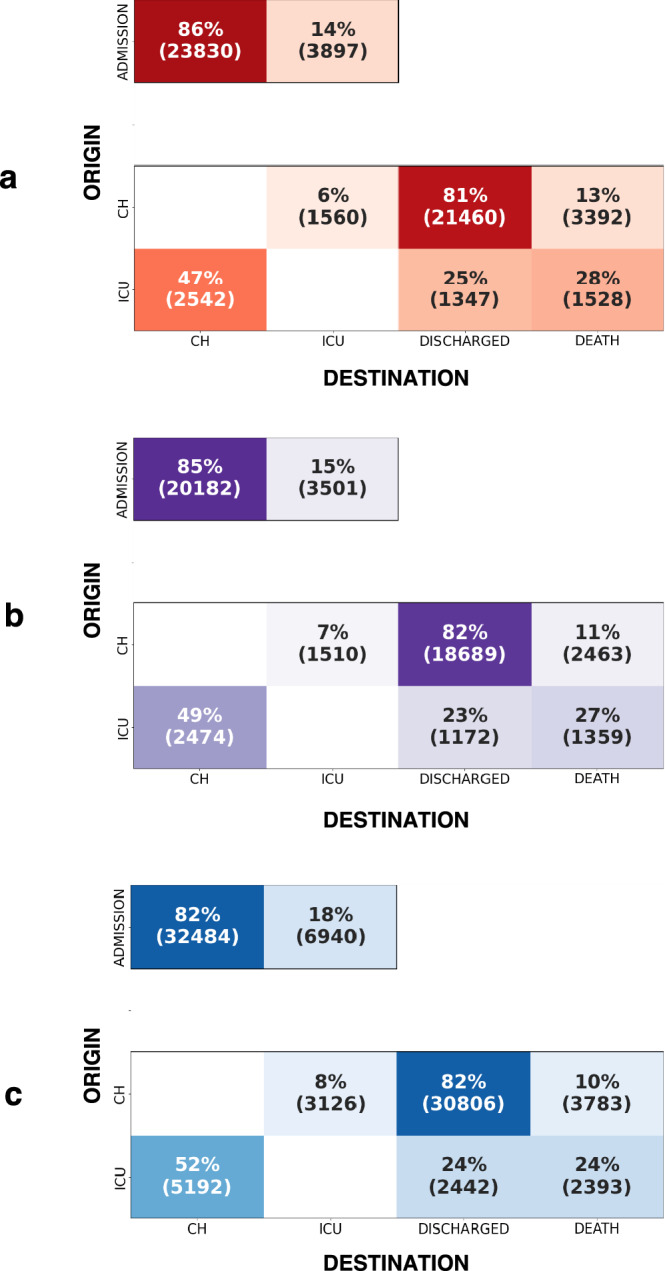
Global transition matrices for each period (or wave). Insets A, B, and C stand for first, second, and third period, respectively. This figure shows the probability of destination given the origin: p(destination|origin). The transition matrices can be read as follows: each box corresponds to the number of stays originating from a particular hospital ward (rows: admission, CH, ICU; total of each row is 100%) and for which transition is made the following day to another hospital ward or state. For example, if we consider all the transitions from CH, 81% of them were directed to discharge.

The transition matrices of the 3 entire periods are shown in Fig. 2. All patients were managed as urgent cases, most of the time with admission to the emergency ward followed by either conventional hospitalization or hospitalization in ICU.

Table 2 shows the main results obtained from the transition matrices. Briefly, when considering stays from CH, the frequency of transition to ICU increased (6% during period 1 versus 8% during period 3) and the frequency of transition to death has statistically decreased between period 1 and 3 (13% during period 1 versus 10% during period 3). This trend is also found for stays from ICU (mortality 28% during period 1 versus 24% during period 3). The proportion of transition to death statistically decreased from 13 to 10% from CH and from 28 to 24% from ICU over the three period.

**Table 2:** Main results obtained from transition matrices.

Origin: CH	Period 1	Period 2	Period 3
	n = 26,412	n = 22,662	n = 37,715
Transition from CH to ICU	1560 (6%)	1510 (7%)	3126 (8%)^‡^
Transition from CH to discharge	21,460 (81%)	18,689 (82%)*	30,806 (82%)^‡^
Transition from CH to death	3392 (13%)	2463 (11%)*	3783 (10%)^‡^

Origin: ICU	Period 1	Period 2	Period 3
	n = 5417	n = 5005	n = 10,027
Transition from ICU to CH	2542 (47%)	2474 (49%)	5192 (52%)^# ‡^
Transition from ICU to discharge	1347 (25%)	1172 (23%)	2442 (24%)
Transition from ICU to death	1528 (28%)	1359 (27%)	2393 (24%)^# ‡^

Data are expressed in number of hospital stays (%).

*CH* conventional hospitalization, *ICU* intensive care unit.

*Significant difference between period 1 and period 2.

^#^Significant difference between period 2 and period 3.

^‡^Significant difference between period 1 and period 3.

**Figure 3:**
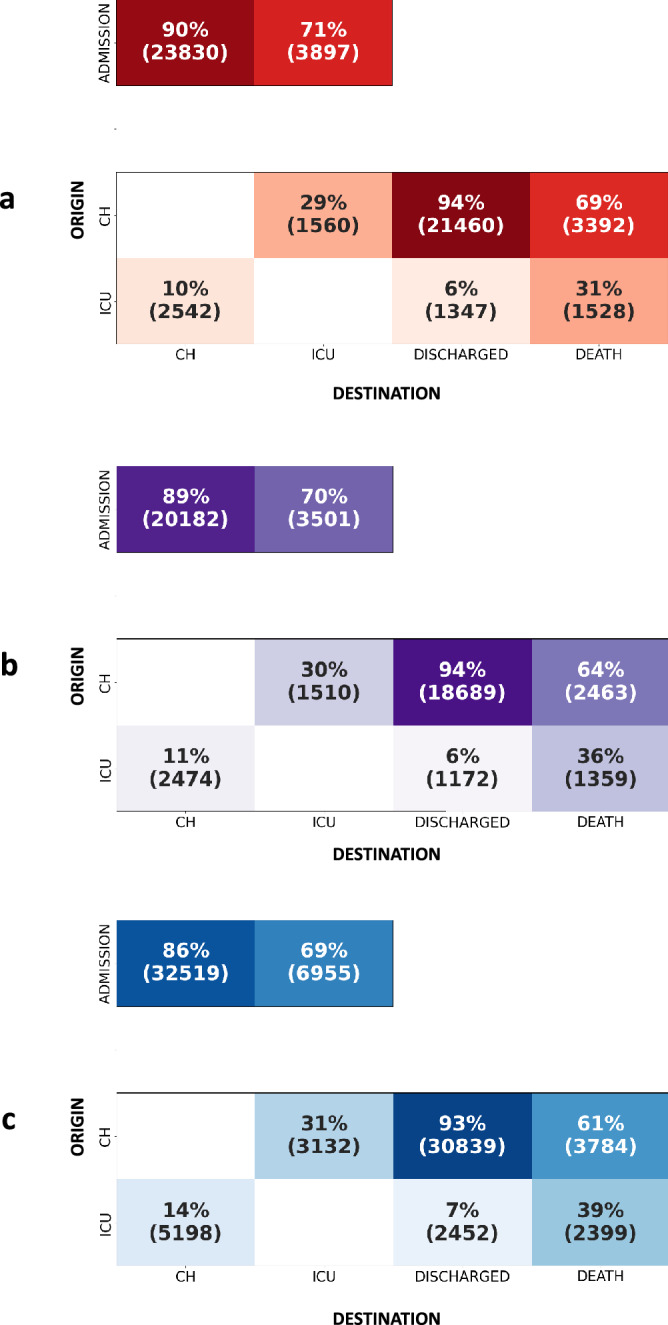
Inverted global transition matrices for each period. Insets (**a–c**) stand for first, second, and third period, respectively. This figure the probability of origin given destination: p(origin|destination). The transition matrices reads as follows: each box corresponds to a percentage (and number) of stays in one hospital ward or state (columns: CH, ICU, discharged or death; total of each column is 100%) and coming from previous hospital ward (rows: admission, CH, ICU). For example: among patients hospitalized in ICU, 29% of them were previously in CH.

The reversed transition matrices are shown in Fig. 3. The frequency with which patients hospitalized in CH coming from ICU was, on average, 10% (Fig. 3a)**.** This frequency statistically increased to 11% during the second period (Fig. 3b) and to 14% during the third period (Fig. 3c). In other terms, in CH, the incoming flow of patients from critical care increased. Among patients who died, the proportion of coming from ICU increased (from 31 to 39%, 1528 and 2399 patients respectively) and as a correlate, the frequency of death among patients coming from CH decreased (from 69 to 61%, 3392 and 3784 patients respectively).

### Analysis by age categories: see Figs. 4 and 5

**Figure 4:**
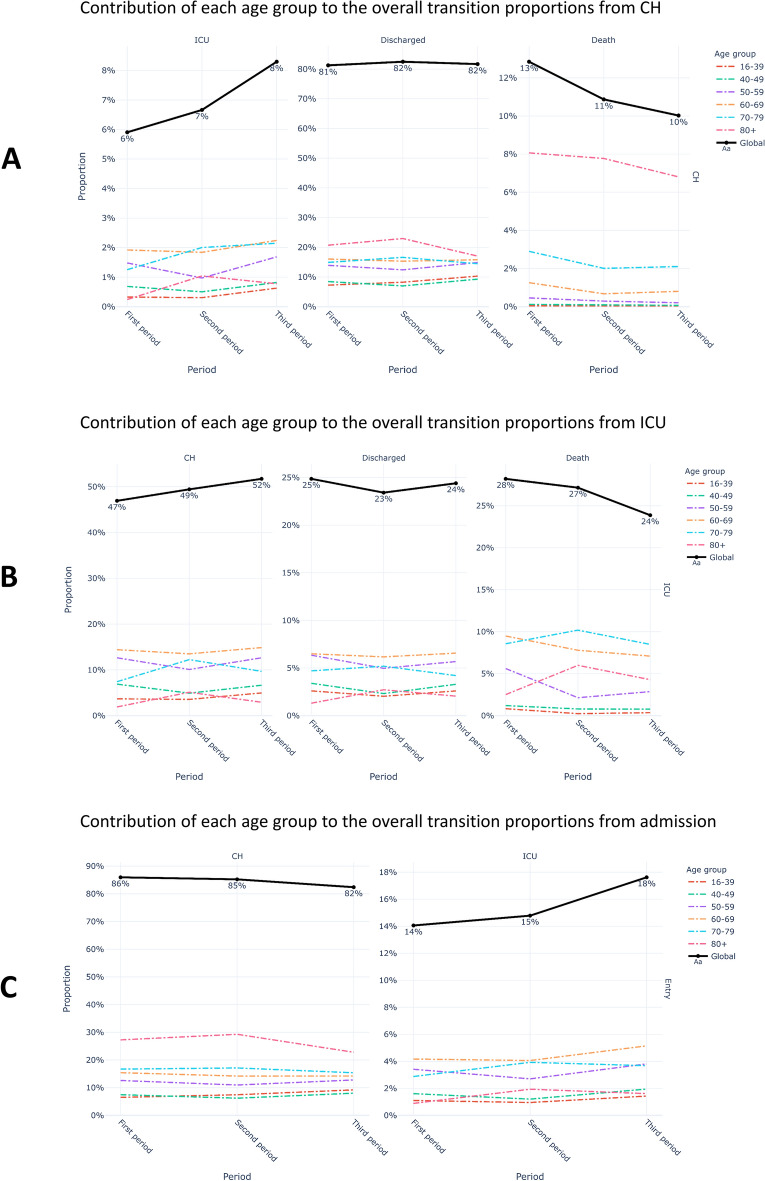
Evolution of the contribution of age in overall transition proportion. (A) From conventional hospitalization, (B) from ICU, (C) from admission.

**Figure 5:**
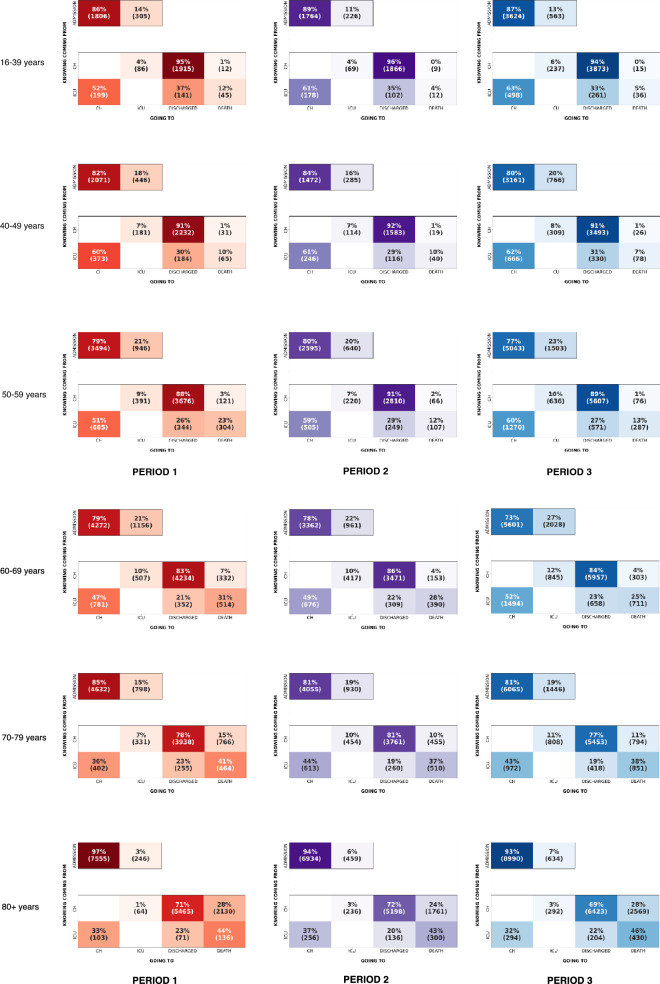
Transition matrices for each period, by age categories.

#### Concerning CH (Fig. 4a)

From period 1 to period 3, the increase in the frequency of transition from conventional hospitalization to ICU concerned all age groups except the extreme high end (80 years and over). On the other hand, all age groups were concerned by the decrease in the frequency of transition to death.

#### Concerning ICU (Fig. 4b)

The increase in transition frequencies from ICU to CH was due to the oldest populations (70 years and older). Despite a steady decrease in transition frequency to death over time, the transition frequency to death among the elderly increased between the first two periods.

### Length of stay (LOS): see Fig. 6a–c

**Figure 6:**
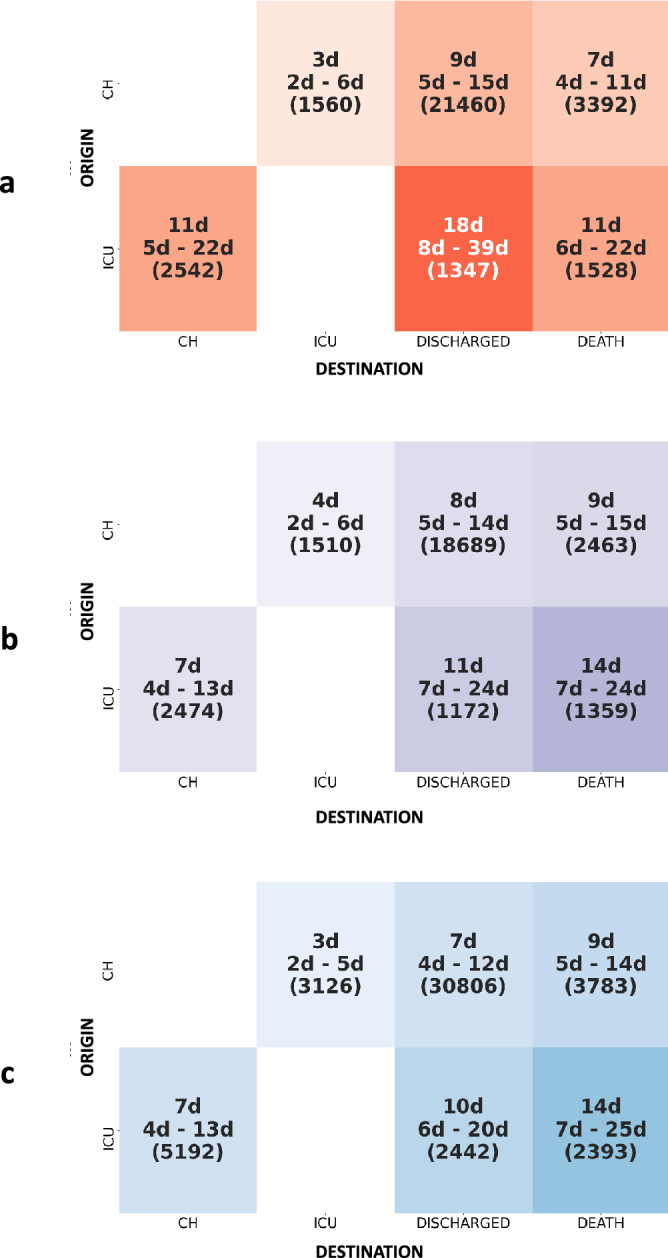
Analysis of lengths of stay by pathway for each of the three periods. Length of stays are expressed in days with their median, 1st and 3rd quartile. Number of patients concerned is given in parenthesis.

During the first period, the median LOS before discharge was 9 days for patients in conventional hospitalization and 18 days for patients in ICU. There was a reduction in LOS between the first and second periods, except for the pathway ending in death. Before death, the median LOS statistically rose from 7 to 9 days for patients hospitalized in CH and from 11 to 14 days for ICU patients. No difference was observed between second and third periods. Before discharge, LOS statistically decreased over the three periods.

## Discussion

We observed that between 2020 and 2021, the COVID-19 epidemic affected Paris area hospitals throughout three identifiable periods, with different repercussions in terms of mortality and care pathways.

Our study compares several waves of the COVID-19 epidemic using the transition matrices method. This method allows us to better observe the variations in intra-hospital care pathways during the different periods. The variations in the frequency of change in hospitalization sector provide elements of comparison for both the severity of the patients' condition and the evolution of care in response to the acquisition of knowledge about the management of COVID-19 patients by healthcare workers.

Since the start of the COVID-19 pandemic, the fear that a more severe variant might develop has been present. While monitoring mutations in the SARS-CoV-2 viral genome can permit quick detection of variants of interest, the effect of these mutations on mortality and hospitalization is difficult to demonstrate^7–9^. Analysis of hospital pathways can provide additional insights into the severity of COVID-19^10^. By analyzing the frequency of death, we are able to identify decreasing severity over successive waves, in particular between waves 1 and 3. From critical care, the frequency of transition to death decreased from 28 to 24% between wave 1 and wave 3. The likelihood of death from conventional hospitalization also decreased.

Nevertheless, it is difficult to assess variant impact using this method. The proportion of stays beginning with an ICU hospitalization has increased (from 14 to 18%) at the expense of stays beginning with a conventional hospitalization (from 86 to 82%). This can be a marker of severity requiring faster management with intensive care. It may also be a sign of better identification and orientation of the most serious progressive forms of COVID-19 over time^11,12^. On the other hand, the dynamic of the epidemic waves and the evolution of hospitalization capacities over time probably had an effect on access to intensive care and therefore on deaths^13^. In our study, this element is supported by the finding of a higher frequency of mortality during the ascending phases of the waves. The implementation of a regional bed management system and the creation of the modular ephemeral resuscitation concept have made it possible to reduce the pressure on bed occupancy^14–17^. These developments have certainly allowed better direct access to critical care, especially for the elderly.

During the early phases of a pandemic, Public Health officials must make decision and design interventions with little epidemiological evidence and given a high degree of uncertainty. In such context, the development of decision support tools for simulating scenarios has become a key process for anticipating and coordinating the adaptation of the response of the healthcare offer^18^. To accomplish such task, Markov chains behaves well when calibrated on a sufficient number of observations and has the advantage to be simple models, and thus easy to understand. A tool based on transition matrices was thus prototyped in the Paris Area in April 2020 to provide rough bed requirement estimation for COVID and non-COVID activities (unscheduled and scheduled activity) at short term (STEP project—Saturation bed anticipation)^19^. Developing a tool to anticipate bed saturation during the COVID-19 crisis has become a major issue in several countries^20–22^. This tool has allowed in a context with no prior information on COVID-19 to help to implement strategy that guides public policy, especially government measures in place for crisis management. Currently, this approach has been replaced in the STEP project by machine learning methods more able to capitalize on the past dynamics of COVID-19 to forecast bed needs and hospital saturation. Because it has led to a constant and observed improvement in performance, the STEP tool has been integrated into the strategy that guides public policy, especially government measures in place for crisis management^23^.

In addition, the analysis of the transition matrices provides a measure of the impact of the COVID-19 vaccination campaign, which began in France in January 2021 (before the 3rd period). Between January and April 2021, vaccination first concerned the over 85 s, then the over 75 s and was then gradually extended to younger age groups. The effects of this strategy can be appreciated on the contribution of each age group to the overall transition proportions from CH, ICU or admission (Fig. 4). The fall in the proportion for the over 80 s between the second and the third period highlights the positive impact of the chosen strategy.

**Figure 7:**
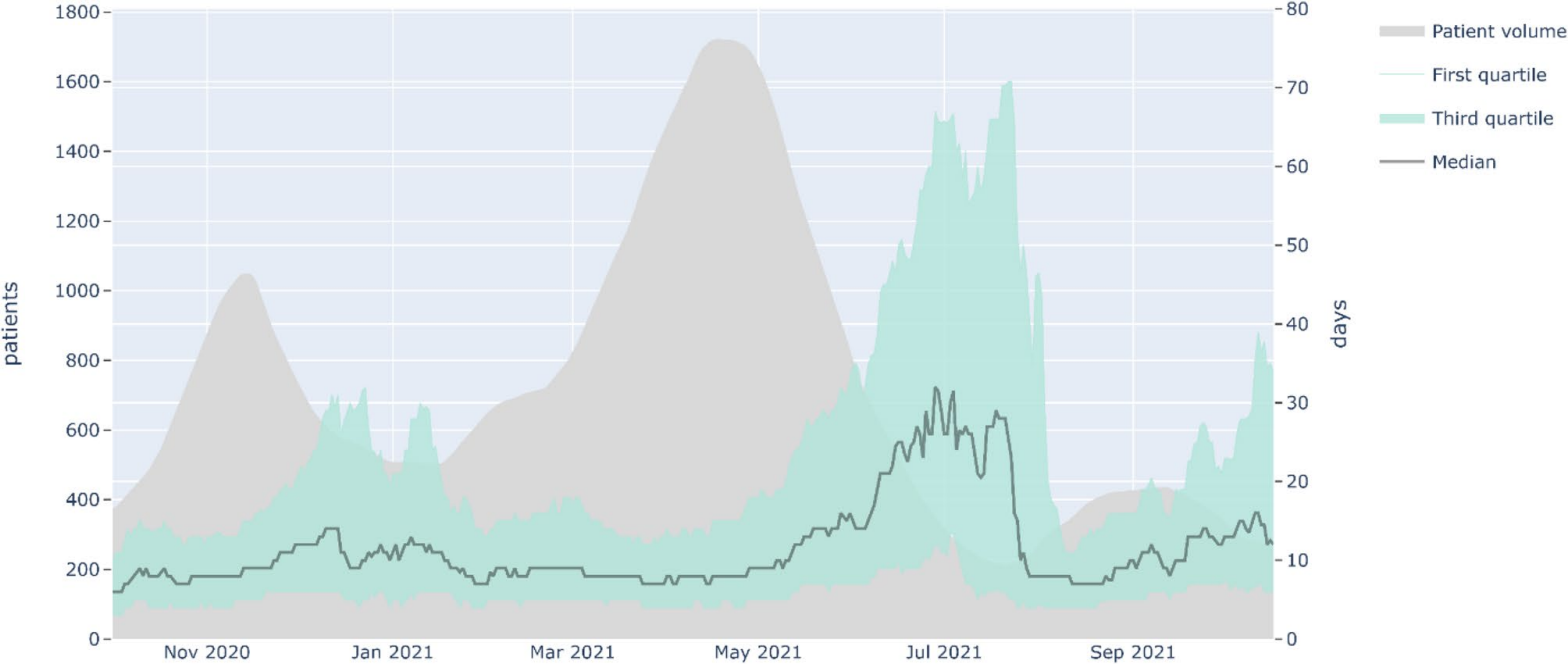
Evolution of the median length of stay in ICU during the 2nd and 3rd periods.

Our study has several limitations. First, the analysis was not done on the whole French territory: the data concerns only the Paris area, which has been strongly impacted by the epidemic and whose population of 12 million represents around 20% of the French population. Second, the data are essentially administrative and contain very few medical elements. It was difficult to describe the patients' clinical state more precisely, in particular the clinical severity. We did not explore the clinical factors explaining the transitions between the different hospital sectors. Nevertheless, an analysis of the data from hospital activity obtained by the French national administrative database (PMSI) will be forthcoming. Third, the pathway analysis did not take into account the daily availability of hospital beds, although this factor can play a determining role in admission type and length of stay. On this point, we indeed noted that median length of stay evolved with epidemic periods: they vary inversely with the volume of patients admitted, especially in ICU. Since October 2020, whether in ICU or conventional hospitalization, a median length of stay of 5 days seems to have been the lowest recorded value. Moreover, when bed occupancy was high, the variability (i.e., interquartile range) decreased: more patients had a length of stay close to the median (Fig. 7). This phenomenon could be due to a decrease in bed availability.

## Conclusion

The profile of hospital pathways of patients treated for COVID-19 evolved significantly during the three first waves of the outbreak that the Paris area endured**.** The use of transition matrices to analyze these pathways is innovative and can provide a broader description of hospital stays for modeling and prediction applications.

## Supplementary Information

Supplementary Information.

## Data Availability

In the case of a request, the data provider may deliver non-identifying aggregated data used for the study, but may not deliver data that could be re-identifying or that are not directly linked to the study or, more broadly, to the objectives of use declared by decree (Article 14 of Decree No. 2020–551 of May 12, 2020 on information systems mentioned in Article 11 of Law No. 2020–546 of May 11, 2020). For any request, please contact arnaud.foucrier@ars.sante.fr.

## References

[1] https://drees.solidarites-sante.gouv.fr/publications/les-dossiers-de-la-drees/parcours-hospitaliers-des-patients-atteints-de-la-covid-19-de

[2] Cummings MJ (2020). Epidemiology, clinical course, and outcomes of critically ill adults with COVID-19 in New York City: a prospective cohort study. Lancet.

[3] Zhou F (2020). Clinical course and risk factors for mortality of adult inpatients with COVID-19 in Wuhan, China: A retrospective cohort study. Lancet.

[4] Druais S (2017). Comparaison des bénéfices médico-économiques des antipsychotiques dans la prise en charge de la schizophrénie en France. L’Encéphale.

[5] Zhang Y, Padman R, Patel N (2015). Paving the COWpath: Learning and visualizing clinical pathways from electronic health record data. J. Biomed. Inform..

[6] Aspland E, Gartner D, Harper P (2021). Clinical pathway modelling: A literature review. Health Systems.

[7] Phan T (2020). Genetic diversity and evolution of SARS-CoV-2. Infect. Genet. Evol..

[8] Toyoshima, Y., Nemoto, K., Matsumoto, S., Nakamura, Y. & Kiyotani, K. SARS-CoV-2 genomic variations associated with mortality rate of COVID-19. *J Hum Genet* **65**, 1075–1082 (2020). 10.1038/s10038-020-0808-9 10.1038/s10038-020-0808-9 PMC7375454 32699345

[9] Young, B. E. *et al.* Effects of a major deletion in the SARS-CoV-2 genome on the severity of infection and the inflammatory response: an observational cohort study. *The Lancet* **396**, 603–611 (2020). 10.1016/S0140-6736(20)31757-8 10.1016/S0140-6736(20)31757-8 PMC7434477 32822564

[10] Lefrancq, N. *et al.* Evolution of outcomes for patients hospitalised during the first 9 months of the SARS-CoV-2 pandemic in France: A retrospective national surveillance data analysis. *The Lancet Regional Health - Europe* **5**, 100087 (2021). 10.1016/j.lanepe.2021.100087 10.1016/j.lanepe.2021.100087 PMC7981225 34104903

[11] Gupta S (2020). Factors associated with death in critically ill patients with coronavirus disease 2019 in the US. JAMA Intern. Med..

[12] Fernandes, F. T. *et al.* A multipurpose machine learning approach to predict COVID-19 negative prognosis in São Paulo, Brazil. *Sci. Rep.* **11**, 3343 (2021). 10.1038/s41598-021-82885-y. 10.1038/s41598-021-82885-y PMC7870665 33558602

[13] Guillon, A. *et al.* Case fatality inequalities of critically ill COVID-19 patients according to patient-, hospital- and region-related factors: a French nationwide study. *Ann. Intensive Care* **11**, 127 (2021). 10.1186/s13613-021-00915-4. 10.1186/s13613-021-00915-4 PMC8375279 34410543

[14] Leroy C (2020). Retour d’expérience sur la cellule régionale d’appui à la régulation des lits de réanimation Covidréa pendant la crise Covid-19. Ann. Fr. Med. Urgence.

[15] Fischer M-O, Pottecher JL (2020). réanimation éphémère en situation sanitaire exceptionnelle. Anesthésie & Réanimation.

[16] Foucrier, A., Hellmann, R. & Rousseau, A. COVID-19: How the Paris area faced the massive influx of critical patients. *Anaesth. Crit. Care Pain Med.* **39**, 575–576 (2020). 10.1016/j.accpm.2020.07.009. 10.1016/j.accpm.2020.07.009 PMC7834620 32727708

[17] Augsburg. *Anaesthesist* **69**, 717–725 (2020). 10.1007/s00101-020-00830-6.

[18] Pollock, B. D. *et al.* Deployment of an interdisciplinary predictive analytics task force to inform hospital operational decision-making during the COVID-19 pandemic. *Mayo Clin. Proc.* **96**, 690–698 (2021). 10.1016/j.mayocp.2020.12.019. 10.1016/j.mayocp.2020.12.019 PMC7833949 33673920

[19] https://www.iledefrance.ars.sante.fr/lia-au-service-de-la-gestion-de-crise-covid-19-zoom-sur-loutil-step.

[20] Römmele, C. *et al.* Bettenkapazitätssteuerung in Zeiten der COVID-19-Pandemie: Eine simulationsbasierte Prognose der Normal- und Intensivstationsbetten anhand der deskriptiven Daten des Universitätsklinikums. 10.1007/s00101-020-00830-6 PMC7441598 32821955

[21] Baas, S. *et al.* Real-time forecasting of COVID-19 bed occupancy in wards and Intensive Care Units. *Health Care Manag. Sci.* **24**, 402–419 (2021). 10.1007/s10729-021-09553-5. 10.1007/s10729-021-09553-5 PMC7993447 33768389

[22] Jombart, T. *et al.* Forecasting critical care bed requirements for COVID-19 patients in England. 10.1186/s12913-021-06509-x.

[23] Regional Health Agency. COVID-19 regional recommendations : adaptation of the supply of hospital care. (2021). https://www.iledefrance.ars.sante.fr/system/files/2021-02/087_ARSIdF-CRAPS_2021-02-24_Doctrine_Adaptation_Offre-soins-hospitaliere_v2.pdf.

