# Supplementary material for: Transition matrices model as a way to better understand and predict intra-hospital pathways of covid-19 patients
Source: Sci Rep. 2022 Oct 20;12:17508. doi: 10.1038/s41598-022-22227-8 (PMC9584905; doi:10.1038/s41598-022-22227-8)
Supplement: Supplementary file 1 — Supplementary Information. [file 41598_2022_22227_MOESM1_ESM.docx]

**APPENDIX**

The diagram below summarizes the possible flows from a hospital admission (entry) which can be either to conventional hospitalization (CH) or to Intensive Care Units (ICU). The hospital pathway of each patient has been described according to the possibilities of transition between CH, ICU, discharge or death, named state.


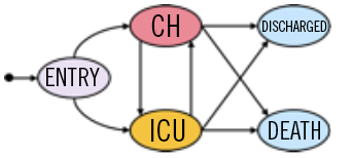


The analysis of the transition matrices was performed over the three periods considered, with a focus on six age categories (16-39 years, 40-49 years, 50-59 years, 60-69 years, 70-79 years and over 80 years)

**Transition matrix construction:**

To build the transition matrices describing the transition frequencies from one state to another, we calculated these frequencies from the data as follows:

1. We selected one state.
2. We set patient counters to 0 for each state.
3. We looped over the set of patients who went to the selected state at least once.
   1. For each patient, the global patient counter is incremented by 1.
   2. We looked at the state following the selected one and incremented the counter associated with this following state by 1.
4. The transition probability is given by:

$$P\left( {State}_{A}\to{State}_{B} \right)=\frac{Number of patients who went from {State}_{A}to {State}_{B}}{Total number of patients in {State}_{A}}$$

*Example*:

We have 100 patients in the "conventional hospitalization” state.

We set counters to 0:

Total number of patients = 0

Number of patients who went to ICU= 0

Number of patients who went to Recovery = 0

Number of patients who went to Deceased = 0

When looping over each patient, we increment these counters whenever a patient goes from state CH to one of the other states.

After looping over each patient, we have the following numbers in the states’ counters:

Total number of patients = 100

Number of patients who went to ICU = 24

Number of patients who went to Discharge = 66

Number of patients who went to Death = 10

Giving us the transition frequencies for the “CH” state row of the transition matrix:

|  | ADMISSION | Conventional hospitalization | ICU | Discharge | Death |
| --- | --- | --- | --- | --- | --- |
| Conventional hospitalization | 0 | 0 | 0.24 | 0.66 | 0.1 |

Since patients cannot go back to their entry state, the associated transition probability is always 0.

Similarly, the diagonal of the transition matrix is 0 because we focus on changing states.

Fitting length-of-stay distribution:

As described, the semi-Markovian model takes two kinds of parameters:

- the transition matrix, whose construction method was described above;
- probability distributions for each state, describing the time spent in these states.

To find these probability distributions, we estimate the parameters of a set of distributions using the maximum likelihood method (or possibly the moments method), based on the observed data. These are mainly Cauchy, chi-squared, exponential, exponential power, gamma, lognormal, normal, power, Rayleigh and uniform laws. For forecasting, these estimated laws are transformed into conditional laws by applying the Heaviside function then a normalization. Thus, the transformed laws are totally asymmetric on the right, allowing predictions. The choice on the candidate laws is carried out by the method of the sum of the square errors.

**Statistical analyses:**

For two populations (waves) of deceased patients with numbers $n_{1}$ and $n_{2}$ and frequencies $f_{1}$ and $f_{2}$, we reject the null hypothesis of equality at $p$ of the probabilities $p_{1}$ and $p_{2}$ at the $5\%$ threshold if

$$\left| f_{1}-f_{2} \right|>1.96\sqrt{p\left( 1-p \right)\left( \frac{1}{n_{1}}+\frac{1}{n_{2}} \right)}$$

where we estimate $p$ by $\frac{n_{1}f_{1}+n_{2}f_{2}}{n_{1}+n_{2}}$.

In the following tables, we will test the fact that $f_{1}$ and $f_{2}$ are significantly different when

$$\frac{\left| f_{1}-f_{2} \right|}{1.96\sqrt{p\left( 1-p \right)\left( \frac{1}{n_{1}}+\frac{1}{n_{2}} \right)}}>1$$

We cannot reject the null hypothesis if the value is less than 1 (and we then say that the values are not significantly different).

The same reasoning applies to the CH, ICU and discharge cases as well as to the numbers of intraregional transfers, types of hospitalization, and outcomes, or for some opposite situations.

The analysis of the mean lengths of stay was done by the Student’s t-test.
